# A Case of Well-Differentiated Papillary Mesothelioma of the Male Peritoneum: Successful Treatment by Systemic Chemotherapy

**DOI:** 10.7759/cureus.1104

**Published:** 2017-03-19

**Authors:** Aziz Bazine, Mohamed Fetohi, Tariq Namad, Asmae Benzekri, Brahim Zainoun, Rachid Tanz, Mohamed Ichou

**Affiliations:** 1 Department of Medical Oncology, Military Hospital Moulay Ismaïl, Meknès, Morocco; 2 Department of Medical Oncology, Military Hospital Moulay Ismaïl, Meknès, Morocco.; 3 Department of Pathology, Nations-Unies Pathology Center, Rabat, Morocco; 4 Department of Radiology, Military Hospital Moulay Ismaïl, Meknès, Morocco; 5 Department of Medical Oncology, Military Hospital Mohammed V, Rabat, Morocco

**Keywords:** well-differentiated papillary mesothelioma, mesothelioma, peritoneal neoplasms, peritoneal effusion, chemotherapy

## Abstract

Well-differentiated papillary mesothelioma of the peritoneum (WDPMP) is a rare subtype of epithelioid mesothelioma, which is usually seen in young women without a history of asbestos exposure, and generally, has an indolent course. The relative rarity of this neoplasm in males prompted us to report this case of a well-differentiated papillary mesothelioma of the peritoneum in a 36-year-old man.

The patient, who had no history of asbestos exposure, presented with abdominal pain and ascites of unknown etiology. Computed tomography showed abundant ascites with nodules of the peritoneal cavity. Laparoscopic examination revealed a large number of white miliary nodules diffusely covering the peritoneum. Pathology revealed a diagnosis of well-differentiated papillary mesothelioma of the peritoneum, based on histomorphology and immunohistochemistry. The patient started chemotherapy with cisplatin and pemetrexed. After six cycles of chemotherapy, the effectiveness of this chemotherapy was checked by only the computed tomography. PET scan was not used because it is not routinely recommended in WDPMP. Few data are currently available in the literature regarding the performance of the PET scan in WDPMP. Nine months later, the patient was free of symptoms.

Based on reviewing the literature and our observations in this case, consultation of other pathologists is highly recommended to discern WDPMP from other disseminated peritoneal diseases, in order to offer the most effective and safe therapeutic strategy. Chemotherapy should be strongly considered if the tumor is unresectable and accompanied by symptoms. Cisplatin and pemetrexed chemotherapy could be a promising therapeutic choice.

## Introduction

Mesotheliomas are relatively rare tumors that arise from the serosal surface of the pleura, peritoneum, and pericardium [1]. Most mesotheliomas of the pleura, peritoneum, and pericardium are either solitary and benign or diffuse and malignant [2]. Diffuse malignant peritoneal mesothelioma (DMPM) occurs mostly in men in the fifth to sixth decades of life, results from occupational asbestos exposure and has a poor prognosis [2-3]. In contrast, well-differentiated papillary mesothelioma of the peritoneum (WDPMP) is a rare subtype of epithelioid mesothelioma, which is generally seen in young women without a history of asbestos exposure and usually has an indolent course [3-4]. Differentiation of this rare tumor from significantly more aggressive lesions, including DMPM and serous papillary carcinomas, can be difficult but is important to avoid unnecessary treatment [5-6]. The relative rarity of this neoplasm in males prompted us to report this case of WDPMP in a 36-year-old man who received systemic chemotherapy.

## Case presentation

The patient is a 36-year-old man with fairly good general health status. There was no history of asbestos exposure or any other significant medical history, and he denied any tobacco or alcohol use. The patient provided written consent for his case to be published.

**Figure 1:**
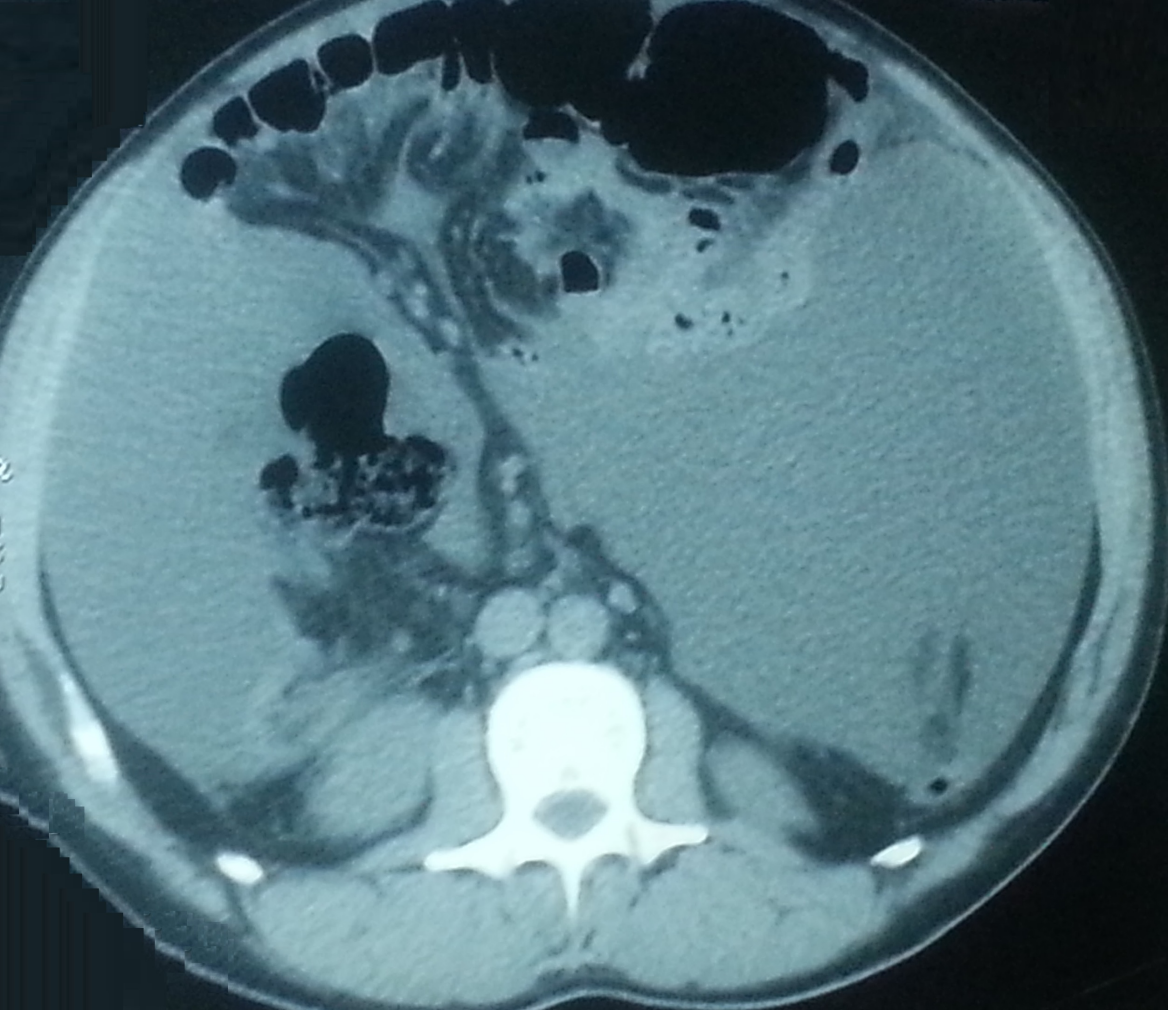
Peritoneal effusion with micronodular peritoneal implants in abdominal computed tomography.

The initial symptoms exhibited by the patient were abdominal pain and an increasing abdominal diameter in the previous two months. A clinical diagnosis of ascites was made. This was confirmed on abdominal ultrasound. Systemic examination was unremarkable. Blood exams showed no abnormalities. Paracentesis was performed removing four liters of yellow, viscous fluid. The ascitic fluid had an elevated total protein (4.7 g/dl) and a low serum-to-ascites albumin gradient (0.5 g/dL). Cytology was negative for malignancy. Bacterial, fungal, and mycobacterial cultures were also negative. Contrast-enhanced computed tomography (CT) of the chest, abdomen, and pelvis revealed a huge amount of peritoneal effusion with micronodular peritoneal implants (Figure 1). Diagnostic laparoscopy was carried out and showed a large number of white miliary nodules of up to 5 mm in size that spread diffusely throughout the parietal layer of the peritoneum. A series of biopsy specimens were taken and six liters of turbid ascitic fluid were drained.

**Figure 2:**
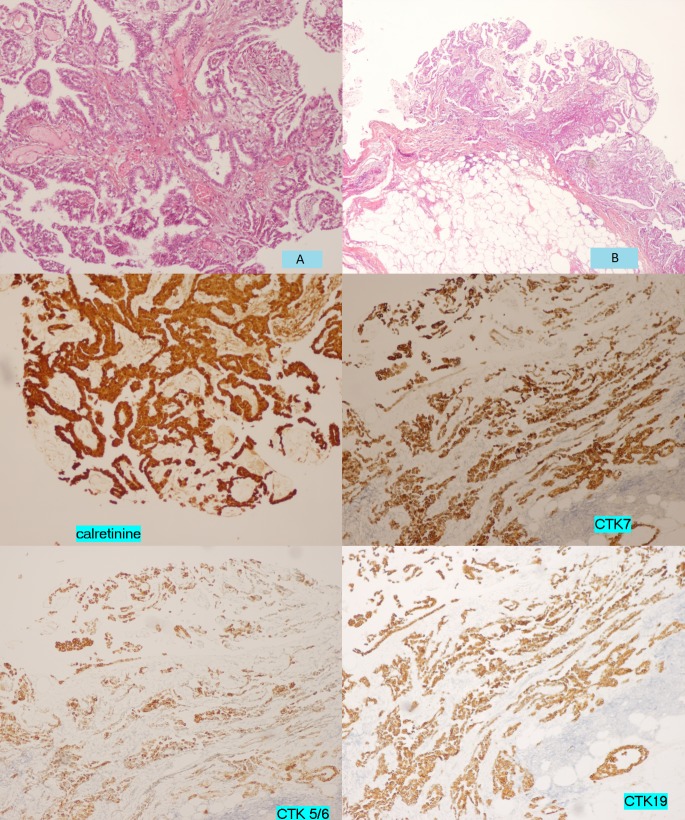
Histologic appearance (A, B) and immunocytochemical analyses of well-differentiated papillary mesothelioma of the peritoneum.

Pathohistological examination of the peritoneal nodules showed a well-developed papillary growth pattern without solid or trabecular areas. The papillae were lined by a single layer of uniform cuboidal cells without cytological atypia, covering the fibrovascular core. Very few mitotic figures were seen amongst the tumor cells. There was no evidence of pseudostratification. Only a single focus of microinvasion was present. On immunohistochemical study, the tumor cells were positive for CTK7 (cytokeratin), CTK5/6, CTK19, and calretinin, but they were negative for CTK20 and Cdx2 (caudal-related homeobox transcription factor 2). A diagnosis of WDPMP was made, based on the histomorphology and immunohistochemistry (Figure 2).

**Figure 3:**
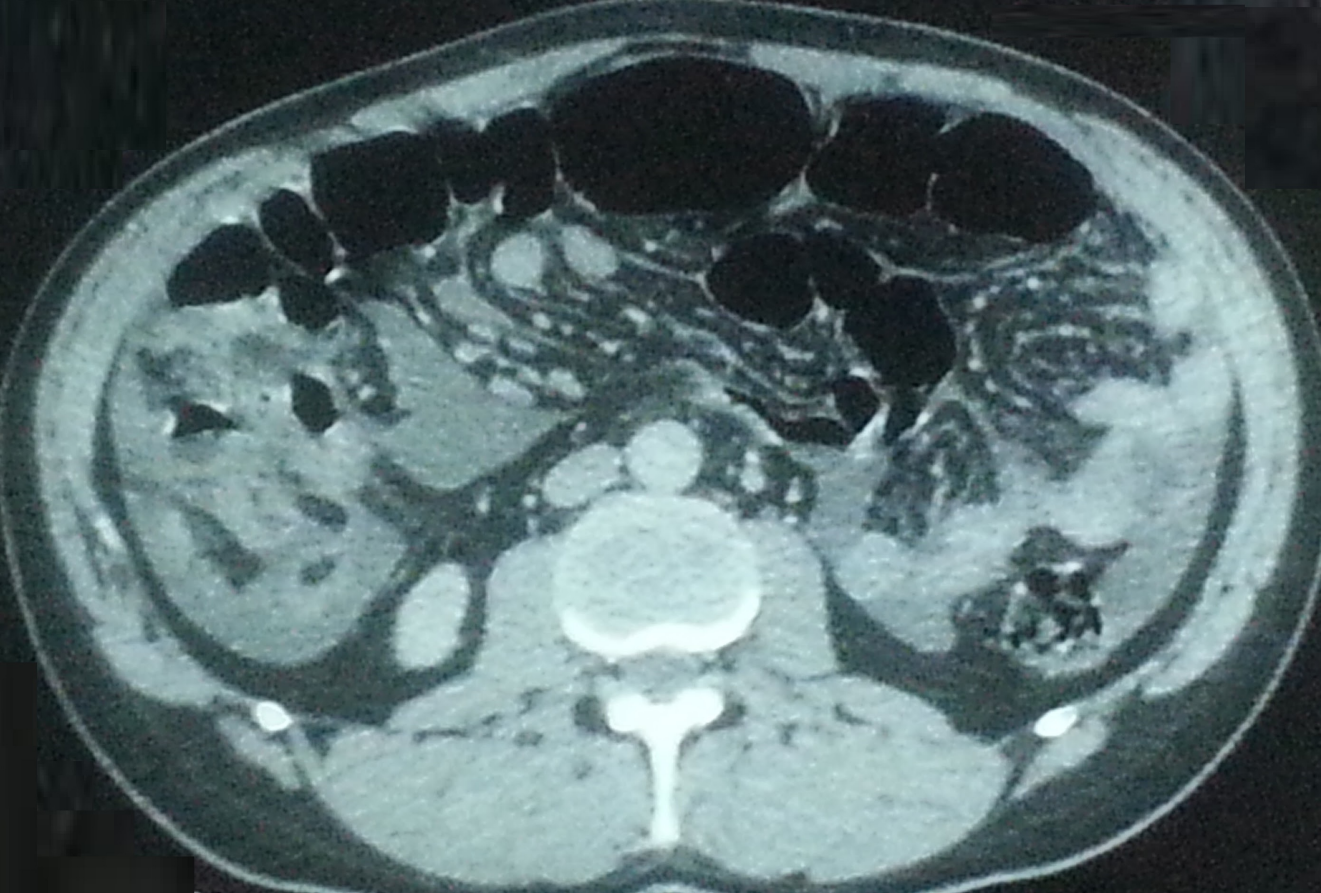
Complete regression of both the ascites and the peritoneal micronodules in computed tomography of the abdomen.

During the pathologic review, paracentesis was performed on multiple occasions for the drainage of ascites. When the diagnosis of WDPMP was confirmed by an external panel of expert pathologists, the patient started chemotherapy with cisplatin and pemetrexed. After six cycles of treatment, he had complete regression of both the ascites and the peritoneal micronodules in the CT control (Figure 3). Nine months later, the patient was still in clinical and radiological remission.

## Discussion

Mesotheliomas are relatively rare tumors occurring from serous membranes. Although commonly found in the pleura, the peritoneum is involved in 20-40% of cases [7]. WDPMP is an extremely rare and distinct type of epithelioid mesothelioma which generally has a relatively indolent course and a far better prognosis than DMPM; however, rare cases with a more aggressive behavior have been reported [4-5, 7]. Most cases affect women of reproductive age in the third and fourth decade. However, sporadic cases also have been described in male patients [4, 6].

The etiopathology of WDPMP is poorly understood. The association with asbestos exposure (or other mineral carcinogens) is not well established. A few cases had a concurrent second neoplasm. These neoplasms included endometrial, renal, ovarian, pancreatic, rectal, colon, and breast cancers. Occasional benign conditions, such as ovarian serous or mucinous cystadenoma, adenomyosis, focal nodular hyperplasia, and teratoma, have also been reported in association with WDPMP. However, no definite and precise aetiology has ever been confirmed [5, 8].

The clinical presentation is insidious, undefined, and unspecific but can appear as a surgical emergency, although the majority of cases were discovered incidentally during surgery for other indications, including adenomyosis, uterine prolapse, small bowel obstruction, ectopic pregnancy, appendicitis, dysphagia, cholelithiasis, and endometriosis. Generally, WDPMP can present with acute and chronic abdominal pain, ascites and distension, weight loss, bloating, menorrhagia, chronic pelvic inflammatory disease, and dyspareunia [5, 7-8]. The ascitic fluid analysis usually shows an exudative process, varying from clear, viscous, or hemorrhagic. No tumor marker is specific and sensitive for diagnosis [7].

WDPMP has been described relatively infrequently in the radiology literature, most likely because of its lack of specific radiological characteristics. CT is clearly superior to ultrasound for the evaluation of WDPMP, especially for the detection of small tumor nodules and for mesenteric or peritoneal thickening. Pelvic involvement may be more adequately assessed by ultrasound. CT may be normal or shows multiple peritoneal nodules, plaque calcification diffusely involving the visceral and parietal peritoneum, peritoneal thickening, omental infiltration, and ascites. Differentiating WDPMP from other disseminated peritoneal diseases, such as tuberculous peritonitis, serous surface papillary carcinoma, malignant mesothelioma, or peritoneal carcinomatosis, is difficult on the basis of CT findings alone. Few data are currently available regarding the performance of the magnetic resonance imaging and fluorodeoxyglucose positron emission tomography/computed tomography [7-9].

Due to no specificity in clinical and radiological features of WDPMP, the diagnostic laparoscopic exam will ensure the definitive diagnosis of this uncommon medical entity. The procedure enables the direct inspection of the abdominal-pelvic peritoneum and intra-abdominal organs and facilitates obtaining biopsy specimens [7]. Laparoscopic findings are generally gray to white, firm, single or multiple nodules of various sizes (0.5 cm to 3.0 cm) which usually involve the parietal and visceral peritoneum, omentum, mesentery, and occasionally, the ovarian surfaces [6].  

Pathologically, coarse or branching papillary architecture with fibrovascular cores is the most commonly seen appearance, with occasional areas of tubulopapillary pattern. The papillae are covered by a single layer of cuboidal or flattened mesothelial cells, with little to no nuclear atypia or mitoses. Invasive foci and psammoma bodies have been described in some cases. Common cytologic characteristics include papillary clusters and minimal cytologic atypia. Immunohistochemistry is used to confirm the diagnosis of WDPMP and differentiate it from other histological similar disease processes, such as serous papillary carcinoma of the ovary and peritoneum, metastatic peritoneal carcinoma, reactive mesothelial hyperplasia, and epithelial malignant mesothelioma with a prominent papillary pattern. Table 1 summarizes the immunohistochemical markers reported in the literature for WDPMP [3, 5-6, 8, 10].

**Table 1:** Immunohistochemical Markers Reported in the Literature for Well-differentiated Papillary Mesothelioma of the Peritoneum

Marker	Number tested	Number positive	Percentage positive
Calretinin	4	4	(100)
HBME-1	1	1	(100)
Alcian blue	6	6	(100)
Cytokeratin	14	13	(93)
PAS	22	18	(82)
Vimentin	4	3	(75)
EMA	6	4	(66)
CEA	9	0	(0)
HMGF-2	4	0	(0)
LeuM1 (CD15)	6	0	(0)
Moc31	1	0	(0)

There are no formal recommendations for treatment of WDPMP. If complete excision is possible, regardless of whether the tumor presents as a single nodule or multiple nodules, the patient should undergo tumor resection followed by close observation, considering the rarity of recurrence after complete resection without adjuvant therapy. In previous isolated reports and small case series, some patients received additional treatment after surgery, including intraperitoneal or intravenous chemotherapy, radiotherapy, immunotherapy, sclerotherapy, and combinations thereof, but these treatments have not shown clear benefits. If the tumor is not completely resectable, chemotherapy should be considered, particularly if the patient is symptomatic and the tumor is extensive. As in the current case, the patient had abdominal pain and abundant ascites requiring drainage by paracentesis on multiple occasions, and the tumor was multifocal and unresectable. The cisplatin and pemetrexed doublet therapy may be an effective chemotherapy for WDPMP. Optimal cytoreduction followed by chemotherapy might be an option, but radical debulking surgery is debatable [4-5, 8].

## Conclusions

On the basis of information that we gathered from the literature, the present case report is one of the few cases that describe extensive and symptomatic WDPMP in a young male patient who was successfully treated with systemic administration of cisplatin and pemetrexed combination, although he did not receive surgery. Given its rarity, we propose that consultation with other pathologists or pathology panels is highly recommended to differentiate WDPMP from other disseminated peritoneal diseases in order to offer the most effective and safe therapeutic strategies based on the disease stages. Chemotherapy should be strongly considered if the tumor is unresectable and accompanied by symptoms; cisplatin and pemetrexed chemotherapy could be a promising treatment choice.
